# Nebulized antithrombin limits bacterial outgrowth and lung injury in *Streptococcus pneumoniae *pneumonia in rats

**DOI:** 10.1186/cc8040

**Published:** 2009-09-09

**Authors:** Jorrit J Hofstra, Alexander D Cornet, Bart F de Rooy, Alexander P Vlaar, Tom van der Poll, Marcel Levi, Sebastian AJ Zaat, Marcus J Schultz

**Affiliations:** 1 Laboratory of Experimental Intensive Care and Anesthesiology (L·E·I·C·A), Academic Medical Centre, University of Amsterdam, Meibergdreef 9, 1105AZ, Amsterdam, The Netherlands; 2 Department of Intensive Care Medicine, Academic Medical Centre, University of Amsterdam, Meibergdreef 9, 1105AZ, Amsterdam, The Netherlands; 3 Department of Anesthesiology, Academic Medical Centre, University of Amsterdam, Meibergdreef 9, 1105AZ, Amsterdam, The Netherlands; 4 Department of Intensive Care Medicine, Vrije Universiteit Medical Centre, De Boelelaan 1117, 1081 HV, Amsterdam, The Netherlands; 5 Department of Medical Microbiology, Academic Medical Centre, University of Amsterdam, Meibergdreef 9, 1105AZ, Amsterdam, The Netherlands; 6 Department of Internal Medicine, Academic Medical Centre, University of Amsterdam, Meibergdreef 9, 1105AZ, Amsterdam, The Netherlands; 7 Centre for Experimental and Molecular Medicine (CEMM), Academic Medical Centre, University of Amsterdam, Meibergdreef 9, 1105AZ, Amsterdam, The Netherlands; 8 Centre for Infection and Immunity Amsterdam (CINIMA), Academic Medical Centre, University of Amsterdam, Meibergdreef 9, 1105AZ, Amsterdam, The Netherlands

## Abstract

**Introduction:**

Disturbed alveolar fibrin turnover is a cardinal feature of severe pneumonia. Clinical studies suggest that natural inhibitors of coagulation exert lung-protective effects via anticoagulant and possibly also anti-inflammatory pathways. Intravenous infusion of the natural anticoagulants increases the risk of bleeding. Local administration may allow for higher treatment dosages and increased local efficacy while at the same time reducing the risk of bleeding. We evaluated the effect of nebulized anticoagulants on pulmonary coagulopathy and inflammation in a rat model of *Streptococcus pneumoniae *pneumonia.

**Methods:**

In this randomized controlled *in vivo *laboratory study rats were challenged intratracheally with *S. pneumoniae*, inducing pneumonia, and randomized to treatment with normal saline (placebo), recombinant human activated protein C (rh-APC), plasma-derived antithrombin (AT), heparin or danaparoid, by means of nebulization.

**Results:**

*S. pneumoniae *infection increased pulmonary levels of thrombin-antithrombin complexes and fibrin degradation products. All nebulized anticoagulants significantly limited pulmonary coagulopathy. None of the agents except danaparoid resulted in changes in systemic coagulopathy. Treatment with plasma-derived AT reduced outgrowth of *S. pneumoniae *and histopathologic damage in lungs. *In vitro *experiments confirmed outgrowth was reduced in bronchoalveolar lavage fluid (BALF) from rats treated with plasma-derived AT compared with placebo. Neutralizing of cationic components in BALF diminished the inhibitory effects on bacterial outgrowth of BALF, suggesting a role for cationic antimicrobial proteins.

**Conclusions:**

Nebulization of anticoagulants attenuates pulmonary coagulopathy during *S. pneumoniae *pneumonia in rats while only danaparoid affects systemic coagulation. Nebulized plasma-derived AT reduces bacterial outgrowth and exerts significant lung-protective effects.

## Introduction

Pulmonary coagulopathy is a hallmark of pneumonia [1-4] and other forms of acute lung injury [2,5,6]. Excessive fibrin deposition within the airways, as a result of activation of coagulation and inhibition of fibrinolysis, compromises pulmonary integrity and function [7,8]. Infusion of recombinant human activated protein C (rh-aPC), one of the natural inhibitors of coagulation, has been shown to benefit patients with severe sepsis or septic shock [9]. Of interest, rh-aPC treatment lead to a more rapid resolution of respiratory failure [9]. In addition, patients with pneumonia as the source of sepsis benefited most from treatment with rh-aPC [10]. Consequently, it has been suggested that anticoagulant and anti-inflammatory effects of rh-aPC in the lungs contribute to better outcome [11,12]. In a recent study in patients with acute lung injury (ALI), systemic rh-aPC treatment did not affect ventilator-free days [13]. However, due to the low number of patients the statistical power to detect a difference in the primary endpoint was limited. Lung-protective effects of antithrombin (AT), another natural inhibitor of coagulation have been demonstrated in a relatively limited number of patients with sepsis [14]. AT did not affect mortality in patients with sepsis in a larger phase III clinical trial but no subgroup analysis on patients with pneumonia as the primary source of sepsis was performed [15]. Heparin is a potent activator of AT and has been used in several preclinical studies to prevent fibrin deposition in models of ALI [2-4]. In a recent study, continuous infusion of low-dose unfractionated heparin did not affect mortality in patients with sepsis [16], nor was mortality affected in a subgroup of patients with pneumonia. However, no subgroup analysis was performed on patients with respiratory failure or ALI/acute respiratory distress syndrome (ARDS).

We recently demonstrated that systemic anticoagulant treatment attenuates pulmonary coagulopathy in pneumonia caused by *Streptococcus pneumoniae *in rats [1]. Intravenously administered rh-aPC, plasma-derived AT or heparin attenuated pulmonary coagulopathy. AT, but not rh-aPC and heparin, exerted significant lung-protective effects in this model. Systemically administered rh-aPC, AT and heparin also attenuated systemic coagulation, which can be considered a major drawback because of increased risks of severe bleeding. We hypothesized local treatment to be equally effective as systemic treatment in attenuating pulmonary procoagulant changes while leaving systemic coagulation unaltered. In addition, we hypothesized that there are beneficial anti-bacterial and anti-inflammatory effects of locally administered plasma-derived AT, as was seen with intravenous administration of this anticoagulant in this model.

## Materials and methods

The Institutional Animal Care and Use Committee of the Academic Medical Center approved all experiments. All animals were handled in accordance with the guidelines prescribed by the Dutch legislation and the International Guidelines on protection, care, and handling of laboratory animals.

### Animals

Pneumonia was induced in male Sprague-Dawley rats (weighing 250 to 300 g; Harlan, Horst, The Netherlands) by intratracheal instillation of 5 × 10^6 ^colony-forming units (CFU) of *S. pneumoniae *(serotype 3, ATCC 6303) as described previously [1].

### Anticoagulant strategies

Rats were randomized to placebo (normal saline) or treatment with 5000 μg/kg rh-aPC (drotrecogin alfa (activated), Eli Lilly, Indianapolis, IN, USA), 500 units/kg plasma-derived AT (plasma-derived AT III, Baxter, Vienna, Austria), 1000 IU/kg heparin (Leo Pharmaceutical, Ballerup Denmark) or 250 anti-Xa E/kg danaparoid (Orgaran, Organon, Oss, the Netherlands; n = 7 per group). Healthy animals without pneumonia served as controls (n = 4). Dosages and timing of study medication were determined using data from previous studies [1,17-19]; the first administration of each agent was 30 minutes before bacterial challenge. Considering its longer elimination half-life (19 to 72 hours [20]) nebulization of AT was repeated every 24 hours; nebulization of saline, rh-aPC (elimination half-life 45 minutes), heparin (elimination half-life approximately 1.5 hours) and danaparoid (elimination half-life 25 hours) was repeated every six hours until sacrifice.

For local administration of saline or study medication we used an exposure system which allows direct exposure of nebulized agents to the noses of the animals. This system consisted of a concentric manifold connected to the necks of bottle-like restraint tubes (CHT 249 restraint tube, CH technologies Inc., Westwood, NJ, USA) in which the animals were confined with their noses adjacent to the bottle necks. The bottles were detachable and the device could be disassembled (e.g., for cleaning) by removing the bottles and removing the manifold. The inhalation chamber was suitable to accommodate several rats at once. One extra outlet was available for measuring the pressure and atmosphere sampling inside the inhalation chamber. The aerosolized agent was supplied to the upper end of the manifold, flows adjacent to the noses of the individual animals, and then was drawn out through the bottom of the manifold. The aerosol atmosphere was generated using the AeronebPro Micropump Nebulizer (Aerogen Ltd., Galway, Ireland). The aerosols were directed to the inhalation chamber by a constant oxygen flow (2 L/min).

### Measurements

At 40 hours after challenge with *S. pneumoniae*, rats were sacrificed and blood samples, bronchoalveolar lavage fluid (BALF) and lung tissue were obtained and analyzed as described previously [1]. For bacterial quantification, BALF and whole blood were plated onto sheep-blood agar plates. Thrombin-antithrombin complexes (TATc; Behring, Marburg, Germany) and fibrin degradation products (FDP; Asserachrom D-Di, Diagnostica Stago, Asnières-sur-Seine, France) were measured in BALF using ELISA. AT, plasminogen activator activity (PAA), and plasminogen activator inhibitor (PAI)-1 activity were measured by automated amidolytic assays [21]. Levels of TNF-α, IL-6 and cytokine-induced neutrophil chemoattractant (CINC)-3 (R&D Systems, Abingdon, UK) and myeloperoxidase (MPO) (HyCult biotechnology b.v., Uden, The Netherlands) were measured using ELISA in lung homogenates.

### Lung histopathology

Histopathology was analyzed and scored by two investigators who were blinded for group identity. Four micrometer sections were stained with hematoxylin and eosin, and analyzed by two researchers blinded for group identity. To score lung inflammation and damage, the entire lung surface was analyzed with respect to the following variables: interstitial inflammation, endothelialitis, bronchitis, edema, pleuritis, and thrombus formation, as described previously [22]. Each variable was graded on a scale of 0 to 4 (0, absent; 1, mild; 2, moderate; 3, severe; 4, very severe). The total histopathology score was expressed as the sum of the scores for all variables.

### *In vitro *studies on the antimicrobial effects of plasma-derived AT

For further analysis of the potential antimicrobial effect of plasma-derived AT we compared cell-free supernatants from BALF of infected animals which were treated with AT (BALF-AT) with BALF from infected placebo-treated rats (BALF-none).

To assess the antimicrobial effect of BALF-AT and BALF-none samples, these samples were added to inoculum suspension and incubated. To assess direct antibacterial activity of plasma-derived AT, inoculum suspension was added to dilutions of plasma-derived AT in phosphate-buffered tryptic soy broth (TSB) medium and incubated.

Innate host immune responses can lead to the expression of cationic antimicrobial peptides (e.g., defensins) [23]. Such peptides can be inhibited by nonspecific binding to negatively charged molecules. We hypothesized plasma-derived AT administration to lead to lower levels of coagulation/inflammation, and therefore to lower concentrations of extracellular protein and coagulation products in the lung, and hence, to less inhibition of cationic antimicrobial peptides. To test whether plasma-derived AT treatment leads to less inhibition of cationic antimicrobial peptides inoculum suspensions were incubated with BALF-AT with or without sodium polyanetholsulphonate (SPS).

### Statistical analysis

All *in vivo *data are expressed as mean ± standard deviation or median with interquartile ranges, as appropriate. Comparisons between the experimental groups and the saline-treated placebo group were performed using one-way analysis of variance (ANOVA) or Kruskal-Wallis test, followed by post-hoc Dunnett's or Dunn's tests, depending on data distribution. A *P *value less than 0.05 was considered statistically significant. The *in vitro *data was log-transformed. Then a one-way ANOVA was performed for separate time points to determine the significance of the difference in growth followed by Bonferroni post-testing. Statistical analyses were performed with SPSS 16.0 (SPSS, Chicago, IL, USA) and Prism 4.0 (GraphPad Software, San Diego, CA, USA).

## Results

### Pneumonia

After instillation of *S. pneumoniae*, typical clinical symptoms of illness (pilo-erection, decreased activity, arched back, decreased food and water intake, and increased respiratory rate) did not occur until about 20 hours later. The animals lost an average of 9% of their bodyweight mainly due to dehydration. Two rats (one in the placebo group and one in the heparin group) died shortly after intratracheal manipulation due to laryngeal edema caused by the procedure. Rats sacrificed 40 hours after the bacterial challenge had evident bilateral macroscopic lung infiltrates.

### Pulmonary coagulation and fibrinolysis

**Figure 1:**
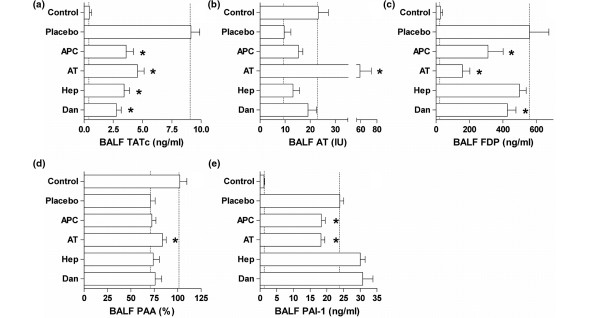
Pulmonary coagulation and fibrinolysis. The effects of anticoagulants nebulized into the lungs of rats on levels of **(a) **thrombin-antithrombin complexes (TATc), **(b) **antithrombin activity (AT), **(c) **fibrin degradation products (FDP), **(d) **plasminogen activator activity (PAA%), and **(e) **plasminogen activator inhibitor-1 (PAI-1) in bronchoalveolar lavage fluid (BALF), 40 hours after intra-tracheal bacterial challenge (*Streptococcus pneumoniae*, serotype 3, ATCC 6303). Dotted lines stipulate the normal values in healthy animals and untreated animals with pneumonia. Data represent mean ± standard deviation. * *P *< 0.01 vs. placebo. APC = recombinant-human activated protein C; AT = plasma = derived human antithrombin; Hep = heparin; Dan = danaparoid.

Bronchoalveolar levels of TATc were increased by pulmonary infection (Figure 1). Rh-aPC, plasma-derived AT, heparin or danaparoid all limited a pneumonia-induced rise of bronchoalveolar TATc (*P *< 0.01 versus placebo). FDP generation was attenuated significantly by all anticoagulants (*P *< 0.01 versus placebo) except for heparin (*P *= 0.26). AT activity seemed to be increased after nebulization of rh-aPC, heparin or danaparoid, although the results were not statistically significant. Administration of AT resulted in supranormal levels of AT (*P *< 0.01 versus placebo and *P *< 0.01 versus control). Pulmonary PAA was significantly reduced with infection, with concurrently enhanced PAI-1 activity in lungs. Both rh-aPC and AT attenuated enhanced PAI-1 activity (*P *< 0.01 versus placebo) and AT treatment significantly increased bronchoalveolar PAA (*P *< 0.01 versus placebo).

### Systemic coagulation and fibrinolysis

**Figure 2:**
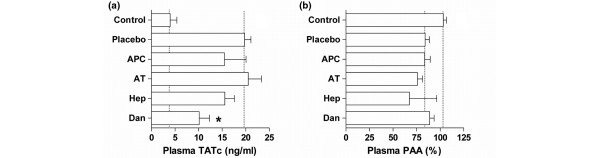
Systemic coagulation and fibrinolysis. The effects of anticoagulants nebulized into the lungs of rats on plasma levels of **(a) **thrombin-antithrombin complexes (TATc) and **(b) **systemic plasminogen activator activity (PAA), 40 hours after intra-tracheal bacterial challenge. Dotted lines stipulate the normal values in healthy animals and untreated animals with pneumonia. Data represent mean ± standard deviation. * *P *< 0.01 vs. placebo. APC = recombinant-human activated protein C; AT = plasma = derived human antithrombin; Hep = heparin; Dan = danaparoid.

Compared with healthy controls, challenge with *S. pneumoniae *caused increased plasma levels of TATc (Figure 2). This was not affected by rh-aPC, plasma-derived AT and heparin. Danaparoid, however, significantly reduced systemic TATc levels (*P *< 0.01 versus placebo). Compared with controls, plasma PAA was significantly decreased after intratracheal challenge with bacteria. None of the treatments altered plasma PAA.

### Bacterial outgrowth from lungs

**Figure 3:**
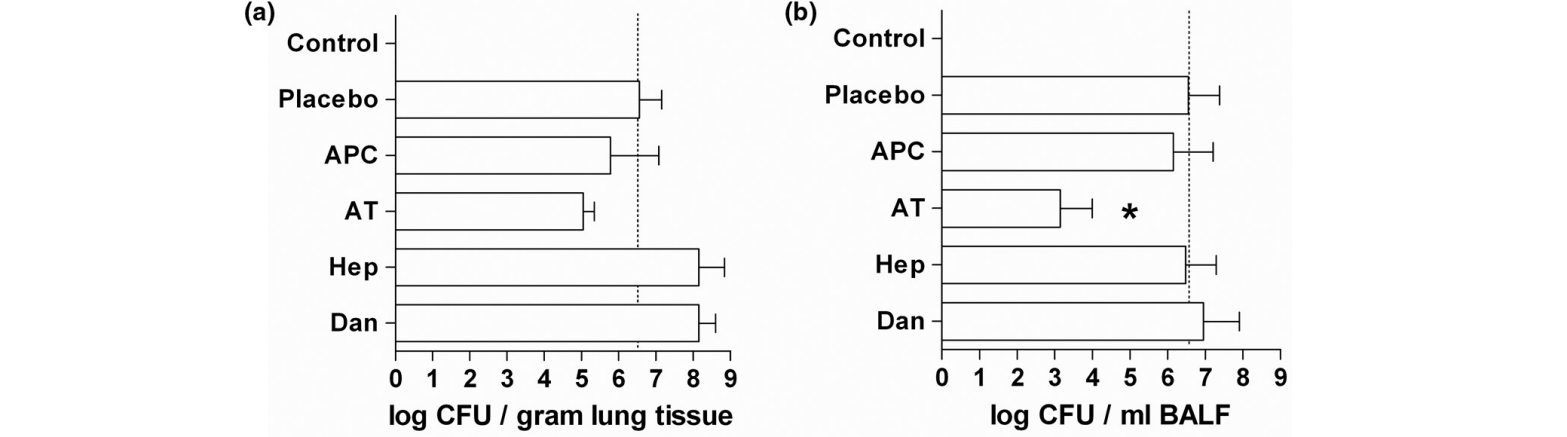
The effects of anticoagulants on numbers of *Streptococcus pneumoniae *colony forming units in **(a)** lung homogenate and **(b)** bronchoalveolar lavage fluid (BALF), 40 hours after intra-tracheal bacterial challenge. Data represent median ± interquartile range. * *P *< 0.05 vs. placebo. APC = recombinant-human activated protein C; AT = plasma = derived human antithrombin; CFU = colony-forming units; Hep = heparin; Dan = danaparoid.

From BALF of rats treated with plasma-derived AT fewer *S. pneumoniae *CFU were cultured (*P *< 0.05 versus placebo; Figure 3). At 40 hours after intratracheal challenge with *S. pneumoniae*, one of six (17%) placebo rats had bacteremia. The proportion of bacteremia was not different in rats treated with rh-aPC or plasma-derived AT, in which two of seven rats (28%) and one of seven rats (14%) developed bacteremia, respectively. The incidence of bacteremia seemed higher, although not statistically significant, in the heparin and danaparoid treated groups (three of seven rats (43%) and five of seven rats (71%), respectively).

### Inflammatory response

**Figure 4:**
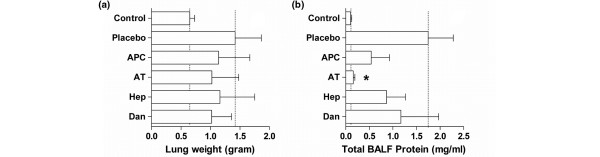
Lung wet weights **(a)** and total protein levels in **(b)** bronchoalveolar lavage fluid (BALF), 40 hours after intra-tracheal bacterial challenge. Dotted lines stipulate the normal values in healthy animals and untreated animals with pneumonia. Data represent median ± interquartile range. * *P *< 0.01 vs. placebo. APC = recombinant-human activated protein C; AT = plasma = derived human antithrombin; Hep = heparin; Dan = danaparoid.

**Figure 5:**
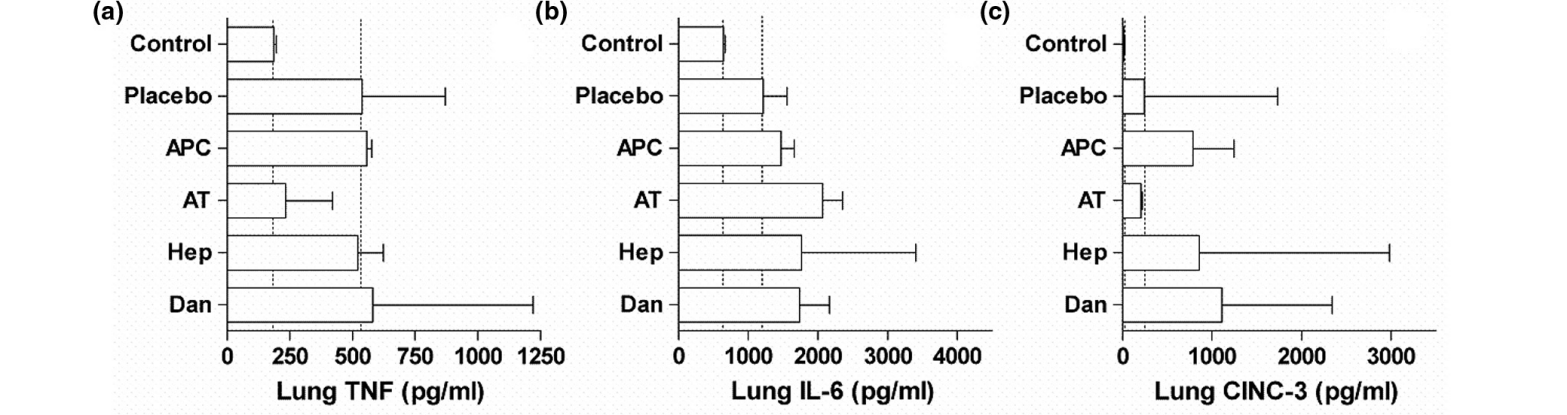
**(a)** TNF-α, **(b)** IL-6, and **(c)** cytokine-induced neutrophil chemoattractant (CINC)-3, determined in lung homogenates 40 hours after intra-tracheal bacterial challenge. Dotted lines stipulate the normal values in healthy animals and untreated animals with pneumonia. Data represent median ± interquartile range. APC = recombinant-human activated protein C; AT = plasma-derived human antithrombin; Hep = heparin; Dan = danaparoid.

After being sacrificed, the weight of the rats' lungs was significantly higher in animals with pneumonia compared with healthy controls (*P *< 0.01; Figure 4). No statistically differences in lung wet weight were observed between the different treatment groups. Total protein concentrations in BALF of rats treated with anticoagulant agents were consistently lower than in rats treated with normal saline. Only differences between plasma-derived AT and saline treatment reached statistical significance (*P *< 0.001 versus placebo; Figure 4). There was an increase in total cell number in the lungs during pneumonia caused by *S. pneumoniae*, mostly attributed to neutrophil influx (Table 1). Plasma-derived AT treatment lead to a decrease of total numbers of cells in the BALF, in particular to fewer neutrophils. MPO activity was not affected by any treatment except for heparin. Lung levels of TNF, IL-6, and CINC-3 were highly variable and there were no differences in pulmonary levels between groups (Figure 5).

**Table 1:** Total cell and neutrophil counts in bronchoalveolar lavage fluid

	Total cells	Neutrophils	MPO
**Controls**	21 (19-24)	0 (0-0)	32 (20-59)
***Streptococcus pneumoniae *pneumonia (t = 40 hours)**			
Placebo	171 (92-206)	73 (39-112)	99 (53-106)
rh-aPC	208 (122-233)	86 (56-104)	186 (66-265)
AT	36 (23-38)*	1 (0-5)**	68 (24-74)
Heparin	417 (313-579)	92 (69-114)	498 (224-932)*
Danaparoid	542 (462-681)	111 (99-125)	407 (167-535)

Cell counts 40 hours after bacterial challenge (*Streptococcus pneumoniae*, serotype 3, ATCC 6303). Controls are uninfected rats (n = 4). Total cell and neutrophil data are expressed as median (interquartile range) × 10^4 ^per milliliter of bronchoalveolar lavage fluid. Myeloperoxidase (MPO) is expressed as pg/mL lung homogenate median (interquartile range). (* *P *< 0.05;** *P *< 0.01 *versus *placebo according to Kruskal-Wallis and Dunn's post-tests).

rh-aPC = recombinant human activated protein C; AT = antithrombin.

### Histopathology

**Figure 6:**
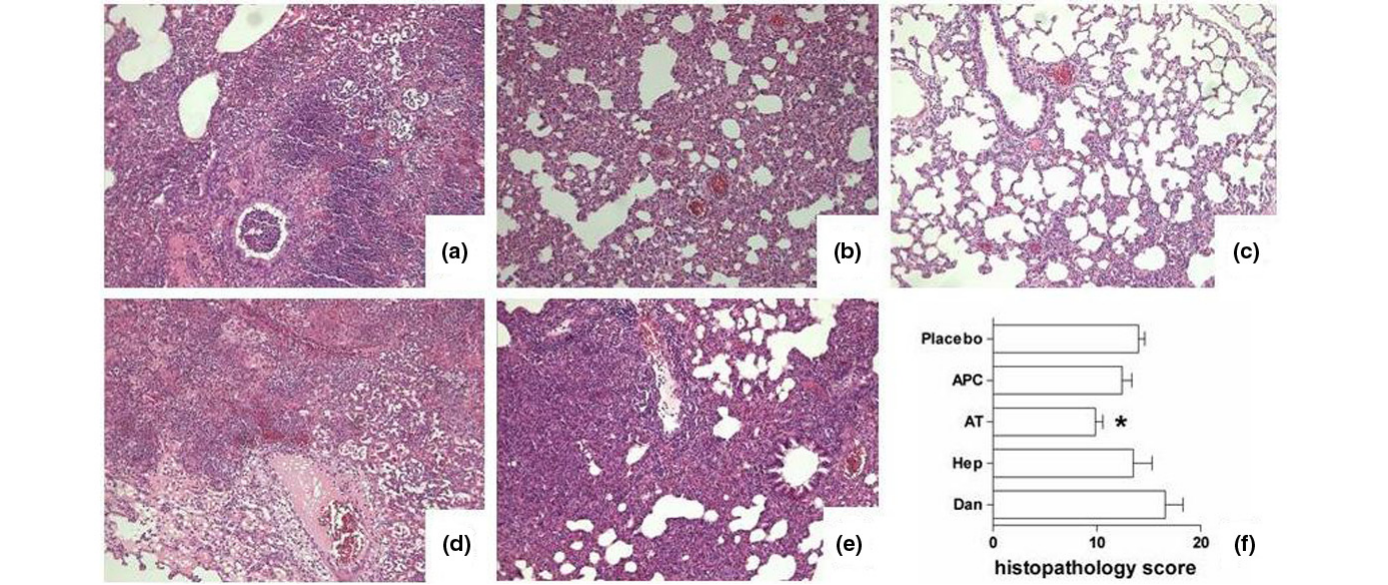
Histopathological changes in *Streptococcus pneumoniae *pneumonia. Shown are representative hematoxylin and eosin-stained photomicrographs (magnification, ×100) of lung tissue from rats treated with **(a) **saline, **(b) **recombinant human activated protein C (APC), **(c) **plasma-derived antithrombin (AT), **(d) **heparin (Hep), **(e) **danaparoid (Dan), at 40 hours after bacterial challenge. **(f) **Total histopathologic scores are presented as median with interquartile range. * *P *< 0.05 vs. placebo.

At 40 hours after induction of *S. pneumoniae *pneumonia, pulmonary histopathology showed dense inflammatory infiltrates, consisting predominantly of neutrophils, localized in the interstitium, alveolar space, and bronchial lumina (Figure 6). Interstitial inflammation, endothelialitis, bronchitis, and edema were present to a variable extent. Only differences between plasma-derived AT and saline treatment reached statistical significance (*P *< 0.05).

### Outgrowth of *S. pneumoniae *in BALF: effect of plasma-derived AT

**Figure 7:**
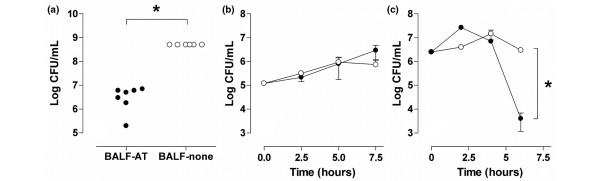
The effects of BALF from placebo treated rats (BALF-none) and BALF from rats treated with plasma-derived AT (BALF-AT) on survival of *Streptococcus pneumoniae in vitro*. **(a) **After five hours outgrowth of *S. pneumoniae *in bronchoalveolar lavage fluid from rats treated with plasma-derived antithrombin (BALF-AT) was reduced (closed symbols), compared with the outgrowth in BALF-none samples (open symbols). * *P *< 0.0001 vs. BALF-AT. **(b) ***In vitro *added plasma-derived AT did not affect the number of colony-forming units (CFU; for clarity only the highest concentrations are shown: 1.1 mg/mL, open symbols; 4.4 mg/mL, closed symbols). **(c) **Sodium polyanetholsulphonate (SPS) blocked the inhibitory effect of BALF-AT on outgrowth of *S. pneumoniae *(BALF-AT without SPS, closed symbols; BALF-AT with SPS, open symbols). Data represent mean ± standard deviation. * *P *< 0.0001 vs. BALF-AT without SPS.

A decrease in bacterial outgrowth was observed when *S. pneumoniae *was grown in BALF-AT samples compared with BALF-none samples after five hours (Figure 7). Adding plasma-derived AT to culture medium had no effect on outgrowth of *S. pneumoniae *in any concentration (i.e., for the entire AT concentration range (0.017 to 4.4 mg/ml) similar bacterial outgrowth curves were observed).

Bacterial numbers in BALF-AT in TSB medium were strongly reduced after six hours compared with the start of the experiment, which confirmed that BALF-AT had an antimicrobial effect on *S. pneumoniae*. Adding SPS to the BALF-AT samples rescued the bacteria, because no decrease in CFU relative to the start of the experiment was seen after six hours.

## Discussion

In this series of experiments we show local anticoagulant treatment through nebulization of rh-aPC, plasma-derived AT, heparin or danaparoid to attenuate pulmonary coagulopathy in *S. pneumoniae *pneumonia in rats. Reduction of pulmonary coagulopathy with nebulized rh-aPC, heparin or danaparoid did not affect the course of pneumonia and ALI. However, reduction of pulmonary coagulopathy with treatment plasma-derived AT was associated with reduced bacterial outgrowth and pulmonary inflammation in this model. Although local administration of rh-aPC, plasma-derived AT or heparin did not affect systemic thrombin generation, nebulized danaparoid reduced systemic thrombin generation.

### Lung-protective effects of plasma-derived AT

Of the investigated nebulized anticoagulant agents only plasma-derived AT treatment resulted in significant lung-protective effects, with less bacterial outgrowth and less histopathological changes. This finding confirms earlier results with systemically administered plasma-derived AT in the same model [1]. AT is one of the major physiologic inhibitors of coagulation, capable of inactivating thrombin and factors Xa, IXa, and VIIa bound to tissue factor [24]. Severe inflammatory processes result in increased consumption of AT [25]. In a small group of sepsis patients, AT treatment improved lung function [14]. In a larger phase III trial with sepsis patients, AT treatment reduced the prevalence of new pulmonary dysfunction, but patient outcome was unchanged [14]. Unfortunately, outcome of preexistent respiratory failure was neither assessed nor reported.

AT may exert its protective effects through increased prostacyclin-mediated inhibition of cytokines, decreased nuclear factor-kB activation [26], and consequent inhibition of leukocyte activation and migration [27-30]. AT may compete with bacterial toxins for binding on endothelial cell proteoglycans [31], limiting the inflammatory response after bacterial challenge and thereby limiting cell and neutrophil influx into the pulmonary department [32]. Here we show that plasma-derived AT treatment strongly inhibited bacterial outgrowth consequently limiting inflammatory response, neutrophil influx and histopathological changes. It remains unclear whether these effects are associated with prostacyclin formation, interference with bacterial toxins (e.g., pneumolysin), or reduced inhibition of antimicrobial peptides (e.g., defensins) through reduced levels of extracellular proteins and coagulation products but at least it is most likely that there are processes involved that are independent of anticoagulant activity. Additional research is needed to elucidate the mechanisms by which plasma-derived AT affects bacterial outgrowth, inflammatory response and migration of neutrophils into the pulmonary compartment.

Our data suggest that therapy with plasma-derived AT might benefit patients with *S. pneumoniae *pneumonia, but additional pre-clinical studies are needed to examine this hypothesis before this therapy is to be tested in patients. The coagulation system plays an important role in containing infections and certain microorganisms even induce profibrinolytic mechanisms to escape from being contained in a fibrin clot [33]. Coagulation and inflammation are pivotal host defence mechanisms, and interference with these pathways should be performed with great care because this may also have deleterious effects. Indeed, previous preclinical studies have demonstrated interfering with the initial procoagulant response in *Pseudomonas aeruginosa *pneumonia in rats and mice is potentially dangerous [34,35].

### Systemic anticoagulant affects of nebulized anticoagulants

Systemic administration of anticoagulant compounds substantially increases the risk of bleeding. In the clinical trial with rh-aPC in patients with severe sepsis, the incidence of serious bleeding was higher in the treatment group than in the placebo group (3.5% versus 2.0%) [9]. In a randomized controlled trial with AT the occurrence of bleeding events in patients with severe sepsis was even more pronounced, especially when AT was combined with heparin (22.0% versus 12.8%) [15]. In the present study with inhaled anticoagulants, none of the investigated agents, with the exception of danaparoid, affected systemic coagulation suggesting inhaled anticoagulants may reduce the risk of systemic bleeding.

The systemic effects on coagulation of nebulized danaparoid suggest that this agent is leaking from the pulmonary compartment into the circulation after local administration. Danaparoid is a relatively small molecule of 5.5 kDa which is more likely to leak into the circulation as compared with rh-aPC (56 kDa) or plasma-derived AT (58 kDa). Heparin is also a small molecule (8 kDa); however, it did not seem to leak into the circulation in our experiments. This may be due to binding of heparin to pulmonary endothelial cells and alveolar proteins and metabolism by heparinases [36]. A recent clinical study of nebulized heparin in patients with ALI; however, did show systemic effects in the highest concentration used [37], although this dose was up to 40% higher than the dose we used in our animal study.

### Previous studies on anticoagulant strategies for pneumonia

Our results are in line with data from previous animal studies. With the same model we previously showed that systemic administration of rh-aPC, plasma-derived AT or heparin altered bronchoalveolar coagulation [1]. In that study systemically administered heparin, however, did not alter pulmonary coagulopathy. Here we show that all nebulized agents limit pulmonary coagulopathy to a similar extent.

rh-aPC exerts profibrinolytic effects through inactivation of PAI-1 [38]. In our experiments reduction of PAI-1 activity in the rh-aPC-treated rats was, however, not sufficient to influence bronchoalveolar PAA. Interestingly, plasma-derived AT affected PAI-1 activity, as well as bronchoalveolar PAA. To our knowledge, no direct effects of AT have been described that account for such alterations in these fibrinolytic markers. It is possible that these changes are due to the reduction in bacterial load observed in the plasma-derived AT-treated rats.

### *In vitro *experiments

The *in vitro *experiments confirmed, at least in part, the findings of the *in vivo *animal study. Indeed, bacterial outgrowth in BALF from rats treated with plasma-derived AT was reduced when compared with BALF from placebo-treated animals. Our experiment with TSB medium with different concentrations of plasma-derived AT showed this was not caused by a direct antibacterial effect of AT. During infection, innate host immune responses can lead to the expression of cationic antimicrobial peptides [23]. Such peptides can be inhibited by nonspecific binding to negatively charged parts of molecules such as coagulation products and other extracellular proteins. We hypothesize that the reduced bacterial outgrowth seen in our *in vivo *experiments might have been due to the fact that plasma-derived AT reduced total protein levels in the BALF by a factor of 10 compared with placebo-treated rats, thereby reducing the nonspecific inhibitory effects on cationic antimicrobial peptides. Neutralizing these cationic antimicrobial peptides with SPS then should attenuate the possible inhibitory effect of BALF-AT on *S. pneumoniae*. Indeed adding SPS to BALF-AT diminished its inhibitory effect on the outgrowth of *S. pneumoniae *after six hours. This finding implies that the induction of coagulation by *S. pneumoniae *serves to protect the bacteria against cationic antimicrobial components, most likely innate host defense peptides, and thus can be considered a novel virulence mechanism. However, because the present study treatment with rh-aPC resulted in quite similar anticoagulant and profibrinolytic effects, while at the same time not affecting bacterial outgrowth or ALI, other mechanisms than anticoagulation may be responsible for the effects of plasma-derived AT found. Details of these mechanisms are presently under investigation.

### Limitations

Our animal study has some important limitations. First, the chosen dosages for each anticoagulant agent are determined based on data from previous studies and pilot studies combined with the efficacy of our nose-only exposure system, and possibility of dissolving each agent to an acceptable volume for nebulization. Although for all studied agents this approach lead to a significant attenuation of pulmonary coagulopathy, lower dosages may also have been sufficient. However, in order to achieve anti-inflammatory effects higher doses of these agents may be required than required for anticoagulant use.

Second, due to differences between the studied drugs aerosol characteristics may differ between the treatment groups. Third, in our study the agents were first administered before induction of pneumonia. Pre-treatment models are useful when exploring novel approaches and mechanisms, post-treatment models more closely resemble the clinical situation.

The fact that the animals were treated intermittently in 6- or 24-hour intervals is another limitation to our study. Although this limited bronchoalveolar coagulation, continuous administration may have been more effective in order to achieve anti-inflammatory effects. Also, during nebulization the rats may have ingested some of the medication. Plasma-derived AT and rh-aPC are most likely to be inactivated immediately by gastric enzymes. Heparin and danaparoid may be absorbed from the digestive tract in small quantities [39]. During each nebulization the animals were exposed to a constant oxygen flow (2 L/min) for a period of 10 minutes. The difference in treatment frequency between the AT-treated animals and the other groups therefore lead to a difference in exposure to oxygen, which in turn may have affected bacterial outgrowth as demonstrated previously [40]. This oxygen effect, however, cannot, explain the persistant inhibition of bacterial growth *in vitro*.

Another limitation of the study is inherent to the fact that our rat model of pneumonia at best mimics the clinical situation. In our model healthy animals of the same sex, age and weight are challenged with a high dose of viable log-phase bacteria inducing pneumonia over a short period of time in a reproducible manner, and clinical factors, such as antimicrobial therapy, mechanical ventilation, fluid management and other supportive interventions are not accounted for. Despite the limitations of our model, our results are in line with previous investigations [1].

Although the incidence of bacteremia was higher in the heparin- and danaparoid-treated groups statistical significance was not reached. Our study, however, may have been underpowered to detect effects on bacterial dissemination.

A limitation of our *in vitro *investigation is that *S. pneumoniae *has a different behavior *in vitro *compared with *in vivo*. For example, bacteria have been observed to reach competence (i.e. obtain the ability to kill their non-competent siblings) in rich medium *in vitro *in early logarithmic growth, which is not seen *in vivo*, where nutrients are generally limited [41,42]. As the peptide which induces competence, competence stimulating peptide, is cationic, it is not unthinkable that its normal activity is altered or diminished in the presence of SPS in our *in vitro *experiments. This altered or impaired activity of competence stimulating peptide or other cationic signaling molecules might result in a change in the growth curve observed for *S. pneumoniae*.

## Conclusions

In conclusion, pulmonary coagulopathy with pneumonia may allow for interventions aimed at local administration of anticoagulant agents. The effects of rh-aPC, plasma-derived AT and heparin were restricted to the pulmonary compartment, nebulized danaparoid also affected systemic coagulation. AT has a lung-protective effect which seems to be related, at least in part, to an indirect role in reduction of bacterial outgrowth of *S. pneumoniae*.

## Key messages

• In experimental pneumococcal pneumonia in rats nebulizing anticoagulants attenuates pulmonary coagulopathy allowing a higher local dose, while reducing systemic effects.

• AT has significant lung-protective effect which seems to be related, at least in part, to an indirect role in reduction of bacterial outgrowth of *S. pneumoniae*.

• More research is required to evaluate the safety and efficacy of nebulized anticoagulants in patients.

## Abbreviations

ALI: acute lung injury; ARDS: acute respiratory distress syndrome; ANOVA: analysis of variance; ARDS: acute respiratory distress syndrome; AT: antithrombin; BALF: bronchoalveolar lavage fluid; BALF-AT: BALF of infected animals which were treated with AT; BALF-none: BALF from infected placebo-treated rats; CINC-3: cytokine induced chemo chemoattractant-3; ELISA: enzyme linked immunosorbent assay; FDP: fibrinogen degradation products; IL-6: interleukin-6; MPO: myeloperoxidase; PAA: plasminogen activator activity; PAI-1: plasminogen activator inhibitor-1; rh-APC: recombinant human activated protein C; SPS: sodium polyanetholsulphonate; TATc: thrombin-antithrombin complex; TNF: tumor necrosis factor-α; TSB: tryptic soy broth.

## Competing interests

The authors declare that they have no competing interests.

## Authors' contributions

JJH participated in the design of the animal study and the *in vitro *bacterial outgrowth studies and collected and analysed the animal data, performed statistical analysis and coordinated the drafting of the manuscript. ADC participated in collecting and analysing the animal data and participated in drafting the manuscript. BFdeR participated in the design of the *in vitro *bacterial outgrowth studies and performed them and participated in drafting the manuscript. APV participated in collecting and analyzing the animal data, and participated in drafting the manuscript. TvdP participated in the design of the animal study and participated in drafting the manuscript. ML participated in the design of the animal study, coordinated the analysis of coagulation and fibrinolysis markers in the animal samples and participated in drafting the manuscript. SAZ coordinated the design of the *in vitro *bacterial outgrowth studies and participated in drafting the manuscript. MJS participated in the design of the animal study and the *in vitro *bacterial outgrowth studies and participated in drafting the manuscript. All authors read and approved the final manuscript.
